# Hemodynamic and echocardiographic characteristics in severe novel influenza A (H1N1) pneumonia

**DOI:** 10.1186/cc9643

**Published:** 2011-03-11

**Authors:** P Theerawit, Y Sutherasan, T Hongpanat, C Kiatboonsri, S Kiatboonsri

**Affiliations:** 1 Ramathibodi Hospital, Bangkok, Thailand

## Introduction

Even though we found a small proportion of patients with severe H1N1 pneumonia developed multiple organ failure, hemodynamic characteristics are beneficial for optimizing treatment. We thus studied hemodynamics including echocardiographic findings in severe H1N1 influenza pneumonia in a single center.

## Methods

All hemodynamic data were collected from severe H1N1 pneumonia patients admitted to the ICU during year 2009 to 2010. H1N1 infections were confirmed by the RT-PCR technique. These positive results were obtained from respiratory tract specimens.

## Results

We enrolled 18 severe pneumonia patients in this study. The mean arterial pressure was 82.62 ± 13.01 mmHg. Thirteen patients were measured for cardiac output (CO) by thermodilution method whereas the remaining cases were measured by echocardiogram. The average CO in the all patients was 5.81 ± 2.49 l/minute. The mean pulmonary artery pressure was 28.77 ± 7.83 mmHg. The central venous pressure and pulmonary capillary wedge pressure (PCWP) were 12.2 ± 3.56 and 15.46 ± 5.22 mmHg, respectively. The SVRI and PVRI were 1,448 ± 457.10 and 293 ± 168.13 dynes·second/cm^5^/m^2^. The CO was higher in ARDS patients than in non-ARDS pneumonia (6.98 ± 2.25 vs. 3.86 ± 0.69, *P *= 0.002). The PCWP in ARDS patients was 16.08 ± 4.93 that was higher than in the non-ARDS group (11.82 ± 1.01), but no statistical significance was demonstrated. The ejection fraction (EF) was measured in 14 patients. The average EF was 59.79 ± 12.87%. There was only one patient having EF less than 30%. There was no statistic significance found in the EF between the ARDS and non-ARDS groups. The E/a ratio and E/E' were 1.29 ± 0.49 and 8.67 ± 2.25, respectively.

## Conclusions

The novel influenza A (H1N1) severe pneumonia resulted in high CO in the ARDS group. The PCWP in these patients was also higher than that in non-ARDS patients. Due to almost all patients having good left ventricular contraction, the etiology of higher PCWP in ARDS patients might result from some degree of high-output cardiac dysfunction. Thus diuretics may have an important role to improve impaired gas exchange in these patients caused by this severe viral pneumonia with ARDS.
